# Giant Ovarian Tumor

**DOI:** 10.3390/medicina59101833

**Published:** 2023-10-15

**Authors:** Tomasz Kluz, Anna Bogaczyk, Barbara Wita-Popów, Piotr Habało, Marta Kluz-Barłowska

**Affiliations:** 1 Department of Gynecology, Gynecology Oncology and Obstetrics, Institute of Medical Sciences, Medical College of Rzeszow University, Rejtana 16c, 35-959 Rzeszow, Poland; jtkluz@interia.pl; 2 Department of Gynecology, Gynecology Oncology and Obstetrics, Fryderyk Chopin University Hospital, 35-055 Rzeszow, Poland; wita.barbara@gmail.com; 3 Clinical Department of Gynecology and Obstetrics, John Paul’s 2nd Municipal Hospital, 35-241 Rzeszow, Poland; p.habalo@op.pl; 4 Department of Pathology, Fryderyk Chopin University Hospital, F. Szopena 2, 35-055 Rzeszow, Poland; marta.kluz@interia.pl

**Keywords:** ovarian tumor, carcinoma, ovarian epithelial tumor, serous cystadenoma, serous cystadenofibroma

## Abstract

Giant ovarian tumors are rare, as most cases are diagnosed during routine gynecological check-ups or abdominal ultrasound examinations. They are a challenge for gynecologists and surgeons. Diagnosis in such patients is difficult due to the limitations of the medical apparatus. Perioperative management requires specialized anesthetic medical care and is associated with high mortality. The paper presents the case of a 23-year-old woman with a giant ovarian serous tumor, characterized by an enlargement of the abdominal circumference, periodic abdominal pain, irregular menstruation, and infertility. The patient attributed these nonspecific symptoms to obesity; therefore, she was hesitant to schedule a doctor’s appointment. The patient underwent laparotomy, and the cyst originating from the left ovary was removed along with part of the organ. An intraoperative examination was performed. After confirming the benign nature of the lesion, the operation was completed. In our work, we concentrated on the multidisciplinary care of the patient who required enhanced medical care from the internal medicine, cardiology, anesthesiology, rehabilitation medicine, and gynecology specialists. There were no hemodynamic changes in the heart during hospitalization. There were no significant early or late postoperative complications. In this case, we also paid attention to compression symptoms resulting from a giant ovarian tumor and the high risk of intraoperative complications resulting from its resection.

## 1. Introduction

We present the case of a 23-year-old woman who was hospitalized due to an enlargement of the abdominal circumference, periodic pains on the left side of the abdomen, scanty and irregular menses (up to 37 days), and infertility, which prompted the patient to seek help in outpatient care. The patient’s abdominal circumference of 195 cm presented challenges for radiologists due to equipment limitations, for anesthesiologists in managing the patient both before and after surgery, and for surgeons/gynecologists because of operational difficulties. The patient required multidisciplinary team care from the very beginning due to the high perioperative risk and the risk of postoperative complications, including the patient’s death.

The patient’s primary concerns were progressive weakness, low mobility, and worsening flatulence over the past half year. She had no significant medical history. The patient had been struggling with obesity since childhood, she lived with a partner, and was socially isolating herself due to her weight and the deterioration of her health.

Her abdominal circumference prior to the operation was 195 cm. She is 156 cm tall and weighs 182 kg. At the time of admission to the hospital, in the physical examination, the patient was afebrile and in moderately good general condition. Vital signs were as follows: HR—110/min, RR 25/min, BP—180/96 mmHg.

**Figure 1:**
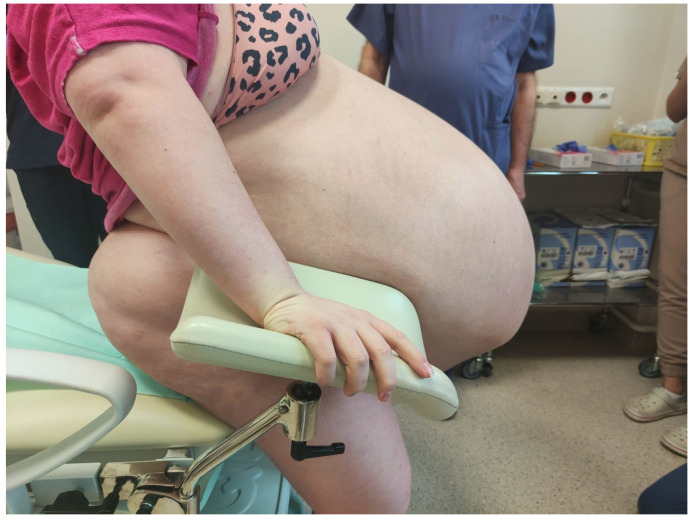
The patient before surgery. Photograph of a 23-year-old woman who presented with respiratory distress and a marked abdominal girth of 195 cm. Side view of the patient’s abdomen before surgery.

**Figure 2:**
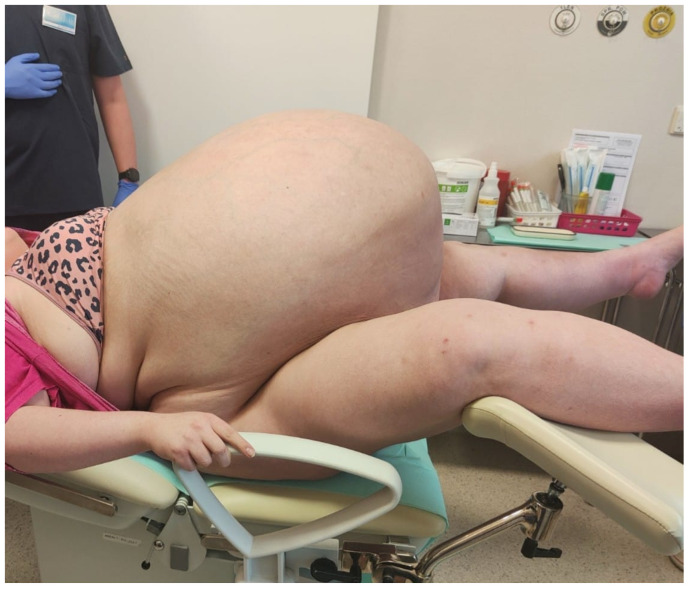
Patient before surgery. Side view of the patient’s abdomen before surgery.

**Figure 3:**
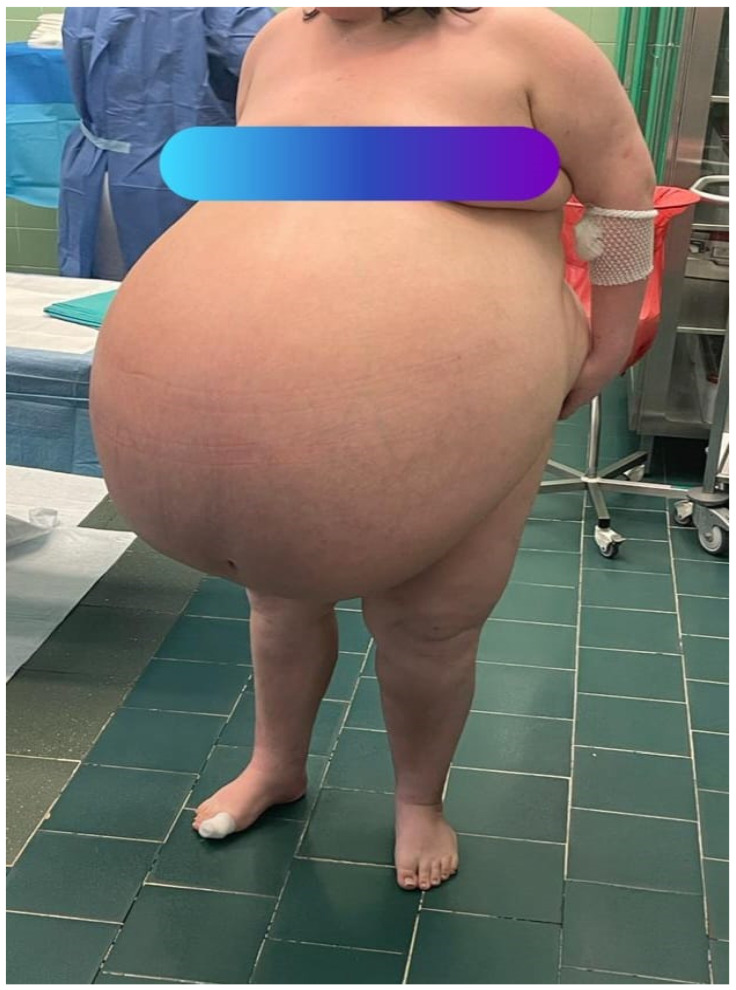
The patient before surgery. Front view of the patient’s abdomen before surgery.

Her abdomen was markedly distended above the chest level, with dilated superficial veins. Additional findings included significant edema of the lower extremities and the sacral region, slightly weakened peripheral pulse in the legs, but no signs of deep vein thrombosis were found. Figure 1, Figure 2 and Figure 3 show the patient before surgery.

In laboratory tests, hemoglobin, electrolytes, and GFR were within the normal range, while urea and creatinine were at the lower end of the range. The coagulation test and gasometry showed no abnormalities. Tumor markers routinely performed in such cases in our clinic (CA-125, HE4, ROMA algorithm, CA-19.9, CA-15.3, AFP, and CEA) were within the normal range. However, the malignant nature of the tumor could not be excluded at that point.

Chest X-ray, except for the limited ultrasound examination, was the only imaging method available, and the findings were as follows: lung fields with normal aeration without focal changes, sharp costophrenic angles, elevation of the domes of the diaphragm with markedly reduced lung volume without effusion, and a large tumor under the diaphragm compressing the abdominal organs. The cardiac silhouette was not assessed.

An abdominal and pelvic ultrasound showed a massive, fluid-filled tumor mass with segmentally visible solid components; however, the origin of the neoplasm could not be reliably identified due to the size of the abdomen of the patient, which was the limiting factor. The ultrasound transducer gave us an insight into the tumor, maximally up to 24–25 cm, which was a small percentage in relation to the diameter of the neoplasm we were dealing with.

Computed tomography and magnetic resonance imaging were ruled out due to the technical limitations of positioning the patient in the apparatus. MRI and CT scans were impossible to obtain in our hospital because the apparatus can only accommodate a patient whose abdominal circumference is below 180 cm, and the patient described in our case report measured above that limit. Hence, such imaging could not be performed.

The patient received and signed a written consent form for the procedure, which included the removal of the uterus and its appendages if the disease was found to be malignant, as well as consent to the creation of a stoma if the disease affected the intestine.

**Figure 4:**
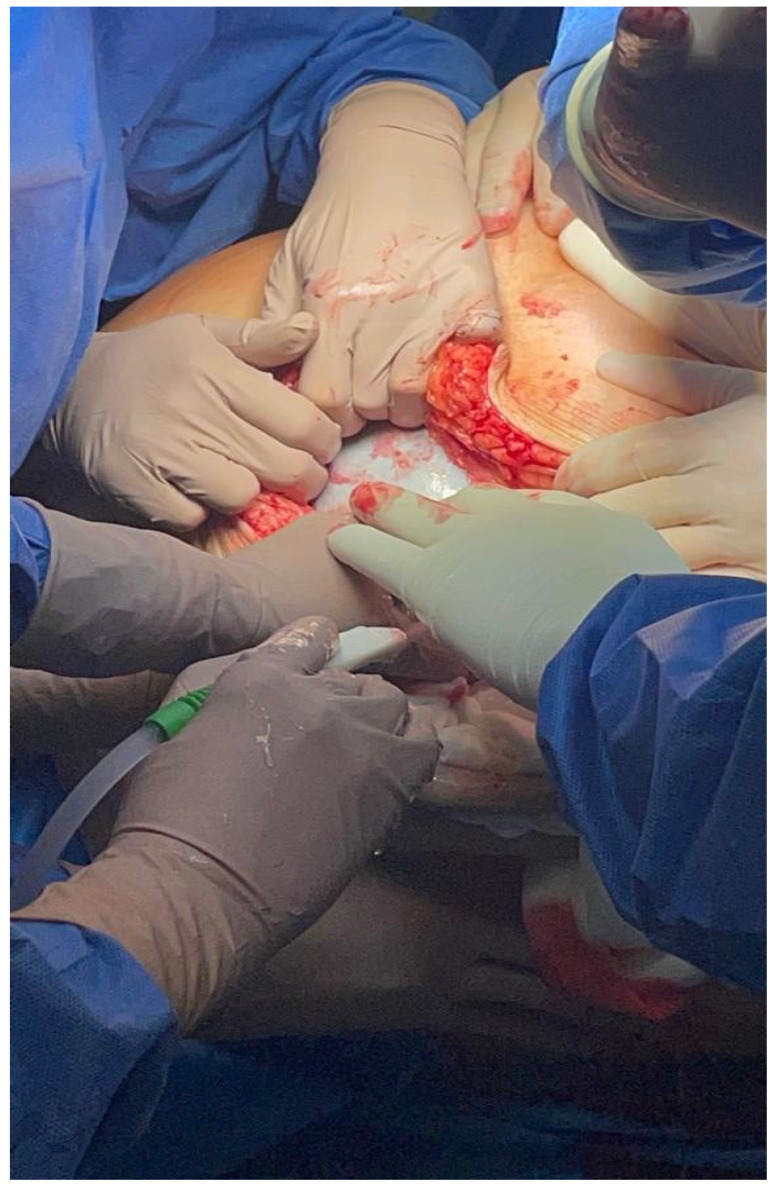
During the operation. Interdisciplinary team during surgery.

**Figure 5:**
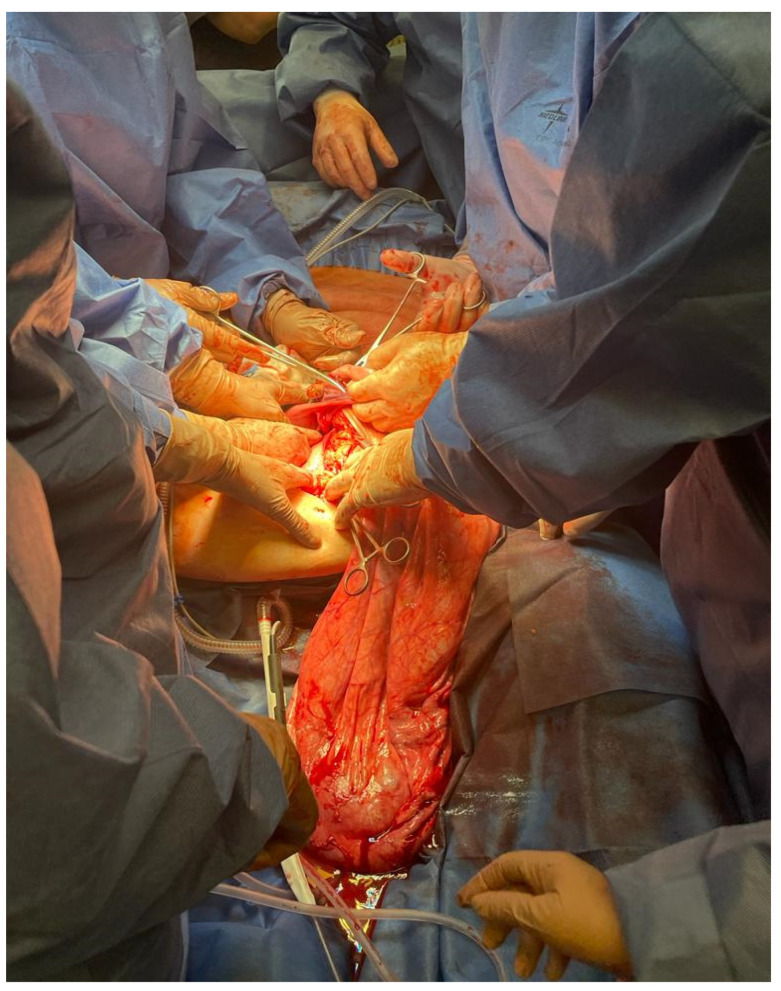
During the operation. Interdisciplinary team during surgery.

**Figure 6:**
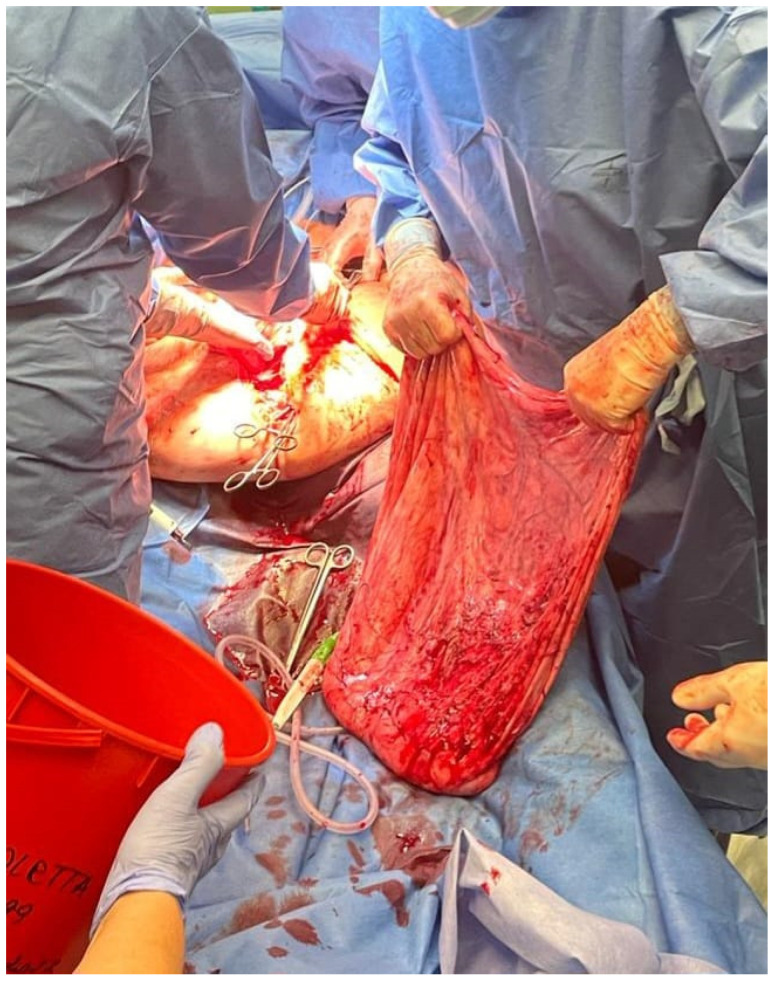
During the operation. Tumor mass removed from the patient.

Shortly after admission to the hospital, the patient underwent laparotomy. Due to extremely challenging operating conditions, the procedure was performed by a multiperson team. Laparotomy was preferred over laparoscopy due to the high probability of the ovarian tumor being malignant. During surgery, the patient was placed under general anesthesia and positioned on the left side to maintain hemodynamic stability. Only in the second part of the operation was the patient placed in the Trendelenburg position because of problems with airway ventilation. Figure 4, Figure 5 and Figure 6 show the patient during laparotomy.

First, access to the peritoneal cavity was obtained through an abdominal incision in the midline below the navel.

**Figure 7:**
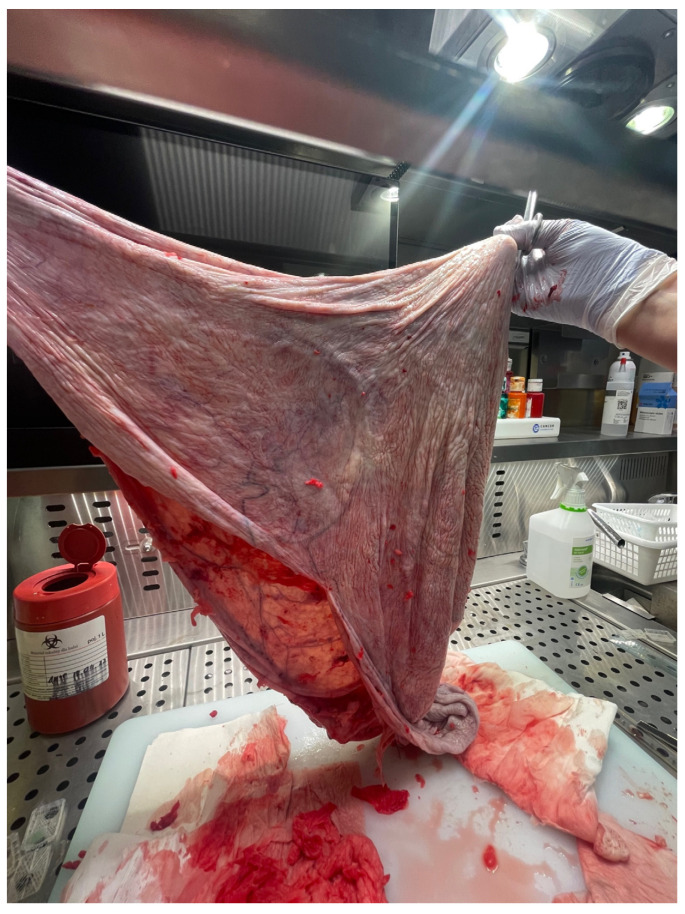
During intraoperative examination. Photo from the pathology department.

After the opening of the abdominal cavity, the tumor wall was incised, and 100 L of fluid was aspirated. Because of its enormous size, the neoplasm could not be weighed after the removal. However, since approximately 100 L of clear, colorless fluid had been drained, it is safe to estimate the tumor mass to be around 100 kg. The abdominal cavity was then inspected, and the adhesions of the tumor wall with the parietal peritoneum and abdominal organs were released. During the operation, it became apparent that the tumor originated from the left ovary. In the next stage, the abdominal and pelvic organs were inspected. The right ovary, right fallopian tube, and uterus were normal, as were other abdominal organs. The tumor wall was then removed along with part of the left ovary. The specimen was sent for a frozen section procedure. During the intraoperative examination, in the gross description, the tumor was reported as an empty, glistening, multiloculated cyst, 80 × 50 × 50 cm, with smooth inner and outer surfaces. The thickness of the cyst wall ranged from 3 to 10 mm. The fallopian tube, measuring 18 cm, was stretched on the surface of the cyst. Figure 7 shows the tumor wall during intraoperative examination.

**Figure 8:**
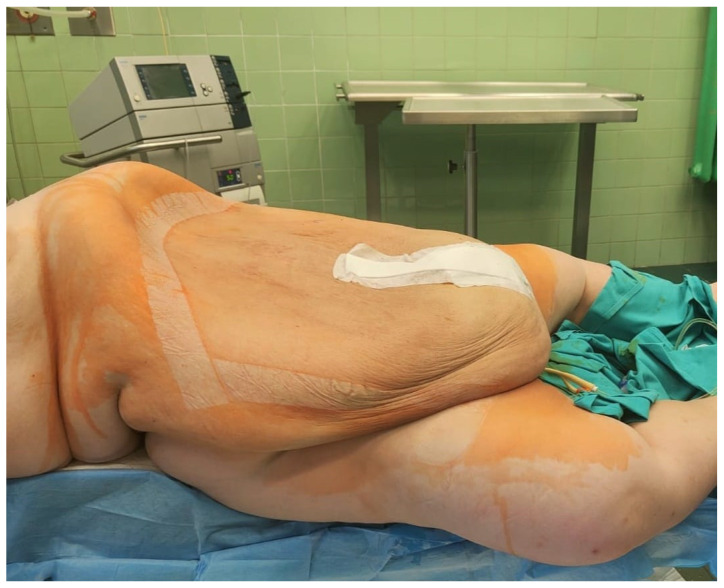
The patient after the operation. Post-operative photo showing the patient’s body parts after removal of the tumor mass.

No macroscopic signs of malignancy were observed. The patient tolerated the surgery well, and no complications occurred during the surgery or under general anesthesia. There was no blood loss or other intraoperative complications, and her recovery was uneventful. The total operation time was 90 min. Figure 8 shows the patient after surgery. Further pharmacological treatment included antibiotic therapy with cefuroxime axetil 3 × 1.5 g i.v. and MTR 3 × 500 mg intravenously, enoxaparin 40 mg/mL, and analgesics. Postoperative recovery was without side effects.

The histopathological report from the intraoperative examination confirmed the neoplasm to be a serous cystadenoma. Considering the patient’s age, nulliparity, and the benign nature of the tumor, the fragment of the left ovary that was not bound to the cyst was preserved in order to maintain the highest possible level of fertility.

Hence, the histopathological report confirmed the specimen obtained during the frozen section to be serous cystadenoma; therefore, there was no need to extend the scope of the operation. After the fixation of the cyst in 10% neutral buffered formalin, numerous sections of the tumor were cut for paraffin blocks, and the definitive histopathological diagnosis was serous cystadenofibroma of the ovary (ICD-O 9014/0). The differences between the intraoperative examination report and the formalin-fixed material result from the fact that multiple samples were taken, not just one as during the frozen section. The differentiating factor is the more prominent stromal component present in the formalin-fixed, paraffin-embedded samples. Nonetheless, this did not change the clinical management of our patient. The sections taken from the fallopian tube showed no pathological findings. The histopathological diagnosis itself is not uncommon; however, the size of the cyst wall was much greater than in everyday clinical practice. 

In addition to the tumor itself, a sample of the fluid from the cyst and peritoneal washing was sent to the department of pathology for examination. Both were fixated with 10% neutral buffered formalin and preserved from clotting with heparin. No malignant cells were found in either of them. In peritoneal washing, mesothelial cells, inflammatory cells and numerous erythrocytes were reported.

In the postoperative period, the patient was under the care of rehabilitation specialists who helped her to recover quickly and improve her physical fitness. The patient started walking the day after the procedure with the help of physiotherapists. The Foley catheter was removed on the second day after surgery. The patient was discharged from the hospital in good condition on the seventh postoperative day. The patient returned to the local clinic 2 weeks after the surgery to have the stitches removed. The patient felt good, the symptoms subsided, she breathed easily and moved efficiently.

## 2. Discussion

The reviewed literature included cases of several patients with giant ovarian tumors, defined as tumors with a diameter of more than 25 cm.

The first reported patient with a giant ovarian tumor was reported by Yamazume et al. in 2007. The described case concerned ovarian cancer. Unfortunately, the patient died 10 h after the surgical removal of the tumor. The patient reported an increasing shortness of breath and mobility problems. The operation was performed with intensive intraoperative monitoring [1]. 

Fu et al. described a case of a postmenopausal woman with a tumor weighing 62 kg. It was the largest tumor ever described in China. Histopathological examination showed that it was a mucinous tumor of borderline malignancy. The authors drew attention to the difficult management of the patient, who struggled with hypotension and postoperative respiratory failure during the operation. These complications were related to the size and weight of the tumor as well as the age of the patient [2].

Pence et al. presented the case of a 63-year-old woman with a large mucinous tumor of borderline malignancy, measuring 35 × 25 × 35 cm in diameter, protruding through an abdominal hernia, complicated by skin necrosis on the surface of the abdominal integuments and perforated by two peptic ulcers. The patient was admitted to the ward in septic shock [3].

Forster described an 87-year-old woman who was hospitalized after a fall. She also had a large abdominal tumor. The patient reported urinary incontinence and abdominal discomfort for several months [4].

Another described patient, aged 58, presented to the emergency department with symptoms of progressive abdominal distension for 8 months. She was diagnosed with a mucinous tumor of borderline malignancy, which measured 44 × 39 × 19 cm [5]. 

The next described patient was 66 years old. She reported to the hospital due to increasing abdominal circumference over 3 years and dyspnea [6]. 

Bamba et al. described a 59-year-old patient with a tumor containing 83 L of fluid. After intubation, the fluid was slowly drained at a rate of 500 mL/min. Then the tumor was removed. There were no serious perioperative complications [7].

In a 20-year-old patient, a case of rapidly disseminating mucous cystadenoma, which reached a diameter of 38 cm, was described. This woman was diagnosed with Cowden’s syndrome [8].

The above examples describe tumors considerably smaller than the one in our case. The largest previously reported case weighed 65 kg. These tumors mainly affect older women in the postmenopausal period. Our patient was 24 years old, her tumor contained 100 liters of fluid, and the circumference of the patient’s abdomen made imaging diagnostics impossible. Computed tomography was not feasible.

Giant ovarian tumors are rare. Most often, they are mucinous tumors. They can sometimes reach very large sizes. They most often occur unilaterally; only about 5% occur bilaterally [9]. However, in our patient, histopathological examination revealed a serous neoplasm. 

Symptoms described in quoted patients as well as in our patient concerned the gastrointestinal tract and included abdominal pain, abdominal pressure, and flatulence. The patients also complained of shortness of breath, which aggravated while lying down and moving. The surgical procedure relies on a multidisciplinary approach to the patient. It is based on surgical, gynecological, and anesthesiological care. It is crucial to pay attention to possible complications in such patients. Appropriate respiratory and cardiovascular management of the patient is of the utmost importance. 

An immense challenge for us was the obesity of the patient, who weighed 182 kg with a height of 156 cm (BMI 74.79). Obesity in the patient is also a challenge for the anesthetist. Airway intubation is often problematic in obese patients, who develop hypoxia within 2–4 min after apnea, even with adequate preoxygenation [10,11].

Possible problems with the circulatory system are associated with hypotension syndrome linked to the compression of large blood vessels by the tumor in the supine position. Removal of the tumor may cause a drop in intrathoracic and intracavitary pressure and subsequent hemodynamic disturbances. To prevent this, Cai proposed slow intraoperative drainage at a rate of 0.5–1 L/min [6]. 

FloTrack sensors, central vein sensors, and echocardiography monitors can be used to prevent cardiovascular problems [12]. 

Another possible complication is intraoperative bleeding. It is necessary to reserve blood for the period of surgery.

Pulmonary edema has also been reported in the postoperative period. It is caused by the sudden reinflation of a previously collapsed or compressed lung. RPE is a rare complication, but associated with high mortality.

Another difficulty in the management of a patient with a giant tumor is postoperative intestinal distension and the possibility of intestinal obstruction. This can be prevented by using abdominal straps and a gastric tube.

Our patient was also treated with analgesics in the postoperative period and physiotherapy.

It is of a great importance not to forget about other possible complications associated with giant tumors, which include hemorrhage, torsion, and rupture.

The tumor may rupture spontaneously or during trauma or pregnancy [13,14]. Tumor rupture is a life-threatening situation due to hypovolemic shock. Tumor rupture can also cause intraperitoneal hemorrhage and peritonitis [15].

## 3. Conclusions

In everyday practice, cystic ovarian tumors are often encountered, but in the modern world, such a huge cyst is rare.

Current imaging techniques can detect even very small ovarian lesions. Unfortunately, these techniques also have their limitations, as we have shown in the case of our patient. In such cases, the possibility of performing an intraoperative examination is of great importance.

In conclusion, a multidisciplinary approach is essential in the care of a patient with a giant ovarian tumor. It is important to anticipate possible complications in order to prevent them properly.

## Data Availability

The data presented in this study are available on request from the corresponding author.
